# Molecular Detection and Genetic Characteristics of Equine Herpesviruses 1 and 4 in Egypt

**DOI:** 10.1155/vmi/9719058

**Published:** 2025-10-10

**Authors:** Emad AL-Ebshahy, Yassien Badr, Ramy E. El-Ansary, Reem Alajmi, Saeed El-Ashram, Alaa Rady, Emad Elgendy

**Affiliations:** ^1^Department of Microbiology, Faculty of Veterinary Medicine, Alexandria University, Abees 10th, Alexandria 21944, Egypt; ^2^Department of Infectious Diseases and Epidemics, Faculty of Veterinary Medicine, Damanhour University, Damanhour, El Beheira 22511, Egypt; ^3^Zoology and Entomology Department, Faculty of Science, Al-Azhar University, Cairo, Egypt; ^4^Zoology Department, Faculty of Science, King Saud University, Riyadh 11451, Saudi Arabia; ^5^Zoology Department, Faculty of Science, Kafrelsheikh University, Kafr El-Sheikh 33516, Egypt; ^6^College of Life Science and Engineering, Foshan University, 18 Jiangwan Street, Foshan 528231, Guangdong Province, China; ^7^Department of Virology, Faculty of Veterinary Medicine, Damanhour University, Damanhour 22511, Egypt; ^8^Department of Microbiology and Immunology, Wake Forest University School of Medicine, Biotech Place, Winston-Salem 27101, North Carolina, USA

**Keywords:** Egypt, EHV-1, EHV-4, equine herpesvirus, phylogeny

## Abstract

The present study investigated the molecular detection and genetic characteristics of equine herpesvirus 1 (EHV-1) and EHV-4 circulating within Egyptian horse populations during 2019–2022. A total of 79 animals were sampled (54 nasal swabs and 25 aborted fetal tissues). PCR assays revealed that 24 (30.3%) and 7 (8.8%) samples were positive for EHV-1 and EHV-4, respectively. Additionally, 5 (6.3%) samples were concurrently infected with both viruses. Four EHV-1 and three EHV-4 isolates were genetically characterized based on partial sequencing of gB gene. The four EHV-1 strains displayed 100% nucleotide identity to one another and to EHV-1 reference strains reported in Egypt and other countries. The three EHV-4 strains were phylogenetically classified into two distinct clusters based on their nucleotide sequences (76%–100% identity). Meanwhile, their deduced amino acid sequences differed by only one amino acid substitution. Our results underscore the critical importance of EHV-1 and EHV-4 as primary contributors of abortion and respiratory illness in horses and highlight the need for further large-scale surveillance and in-depth characterization studies to improve our understanding of these viruses' epidemiology in Egypt and to develop a robust control strategy.

## 1. Introduction

Equine herpesviruses (EHVs) are important ubiquitous pathogens of equids, causing devastating economic, health, and welfare repercussions [1]. They are double-stranded DNA viruses belonging to the Herpesviridae family. To date, nine distinct EHVs have been found in equids, six (EHV-1, 3, 4, 6, 8, and 9) belong to the genus *Varicellovirus* within the subfamily *Alphaherpesvirinae* and three (EHV-2, 5, and 7) belong to the subfamily *Gammaherpesvirinae* [2].

EHV-1 and EHV-4 are genetically and antigenically linked and are regarded as the most important and prevalent viruses in horse populations globally [3]. EHV-1 causes a wide range of clinical symptoms, including respiratory distress, neuropathogenic disorders (myelencephalopathy), abortion in pregnant mares, and perinatal death [4]. Meanwhile, EHV-4 is connected largely to respiratory sickness and infrequently to abortion and neurological abnormalities [5]. Clinically, EHV-1 respiratory disease is virtually indistinguishable from that induced by EHV-4 and other respiratory pathogens [6]. In addition, abortions usually occur in the third trimester of pregnancy and often without premonitory signs [7,8]. EHV-1 and EHV-4, like all other herpesviruses, can form life-long latent infections that can periodically be reactivated, producing recurrent clinical illness and transferring the infection to other hosts [1].

The highly sensitive, specific, and trustworthy polymerase chain reaction (PCR) has shown to be an effective method for quickly identifying and distinguishing between EHV-1 and EHV-4 infections [9,10]. Furthermore, most phylogenetic investigations have focused on the envelope glycoprotein B (gB) gene, which is necessary for viral attachment, entrance, and direct cell-to-cell dissemination, as well as a significant target for host immune responses [11,12].

In Egypt, alphaherpesviruses (EHV-1 and EHV-4) and gammaherpesviruses (EHV-2 and EHV-5) are thought to be endemic [13–15]. However, most economic losses were attributed to EHV-1 and EHV-4 infections [13,14,16]. The ongoing emergence of these viruses exerts a major negative impact on the equestrian industry, especially in the absence of vaccination programs. Thus, there has been a crucial need for continuous monitoring and characterization of the circulating field strains to better understand the epidemiological situation of these viruses in Egypt and to develop a robust control strategy.

The study area, Alexandria province, is one of the densely populated Egyptian regions with horses, in which the incidences of EHV-1 and EHV-4 infections were reported [14]. However, to date, none of these viruses has been genetically characterized. Therefore, the present study aimed to identify and genetically characterize EHV-1 and EHV-4 strains circulating within horse populations in Alexandria province, Egypt, during 2019–2022.

## 2. Materials and Methods

### 2.1. Study Area and Sample Collection

During 2019–2022, a total of 79 horses aged 1.5–10 years were sampled across different stud farms and veterinary clinics in Alexandria province, Egypt. Nasal swabs (*n* = 54) were collected from horses exhibiting respiratory clinical signs, including coughing and nasal discharges. In addition, pooled specimens of the placenta and fetal lung (*n* = 25) were obtained immediately after abortion. The majorities of mares were aborted during the last trimester of pregnancy and exhibited no clinical signs around the time of the abortion. Each sample was immediately transferred into a sterile transport solution containing equal quantities of glycerol and phosphate-buffered saline (PBS), pH of 7.4 before being delivered directly to the laboratory on ice packs, and then kept at −20°C until further processing.

### 2.2. Sample Processing and DNA Extraction

The collected samples were processed using the World Organization for Animal Health (OIE) manual [17]. The QIAamp MinElute Virus Spin kit (Qiagen, Germany) was used to extract total DNA from a 200 μL aliquot of each processed sample. The manufacturer's instructions were followed. DNA was recovered in 50 μL of DNase-free water and kept at −20°C until use.

### 2.3. Pan-herpesvirus-Nested PCR Assay

DNA extracts were subjected to a nested PCR assay targeting highly conserved motifs within the DNA polymerase gene of EHVs. Two consecutive amplification rounds were carried out using EmeraldAmp GT PCR master mix (2× premix; Takara Bio, Japan) and two sets of consensus primers (Table 1), as previously described [18]. The initial amplification mixture contained two forward primers (DFA-F and ILK-F) and one reverse primer (KG1-R). Subsequently, a 2.5 μL aliquot of the first amplification round was used for a second round with the forward primer TGV-F and reverse primer IYG-R. The amplification rounds included an initial denaturation cycle (94°C for 5 min), 45 cycles of denaturation (94°C for 30 s), annealing (46°C for 1 min), and extension (72°C for 1 min), and a final extension step (72°C for 10 min). The amplification products were examined by electrophoresis in a 1.5% (w/v) agarose gel with ethidium bromide stain at a final concentration of 0.5 μg/mL and assessed by UV transillumination.

### 2.4. Conventional PCR for EHV-1 and EHV-4 Detection

DNA samples that had examined positive by pan-HV-nested PCR were subsequently subjected to conventional PCR using primer pairs selected for the gB gene of EHV-1 and EHV-4 (Table 2), as previously described [14,19]. The reaction mixture comprised 12.5 μL of 2× EmeraldAmp GT PCR master mix (Takara Bio, Japan), 6 μL of DNA extract, and 1 μL (20 pmol) of each primer and adjusted to a 25-μL volume with nuclease-free water. Amplification conditions for EHV-1 were as follows: one hold step at 94°C for 5 min for initial denaturation, 35 cycles of denaturation (94°C for 1 min), annealing (55°C for 45 s) and extension (72°C for 1 min), and a terminal extension step at 72°C for 10 min. The thermal profile for EHV-4 was the same as EHV-1, except that the annealing step was done at 66°C for 1 min. PCR products were separated in an ethidium bromide–stained agarose gel and seen by UV transillumination.

### 2.5. Sequencing and Phylogenetic Analysis

The most intense DNA bands, which corresponded to the predicted sizes of the amplified products of the gB gene, were chosen for sequence analysis. The gel slices containing the bands of interest were submitted to DNA extraction and purification employing the QIAquick Gel Extraction Kit (Qiagen) as directed by the manufacturer. The purified fragments were sent to Animal Health Research Institute, Dokki, Egypt, where they were Sanger-sequenced in both forward and reverse directions employing the same amplification primers and a BigDye Terminator v3.1 Kit (PE Applied Biosystems, USA) according to the manufacturer's instructions. The EHV-1 and EHV-4 gB gene sequences were edited and aligned with the corresponding sequences in the GenBank database employing the ClustalW method implemented in MEGA11 software [20]. The aligned sequences were then used to create phylogenetic trees employing the maximum likelihood technique using the Tamura–Nei substitution model and 1000 bootstrap iterations.

### 2.6. Statistical Analysis

Chi-square test was used to test the significance of the detection rates of EHVs in different samples. The SPSS package system was used for all analysis and calculations. A *p* value less than 0.05 was set as the limit of significance.

## 3. Results

### 3.1. Molecular Detection and Typing of EHV

Pan-herpesvirus-nested PCR assay testing for EHV infection identified the presence of EHV DNA in 36 (45.5%) samples from the 79 assayed. The resulting amplicons were approximately 250 bp in size, which is consistent with the expected size range (215–315 bp). The pan-herpesvirus-nested PCR-positive samples were then confirmed as positive by the conventional PCR assay used for EHV typing. As expected, the 869-bp and 508-bp bands were generated by amplification of gB gene fragments of EHV-1 and EHV-4, respectively. A total of 24 (30.3%) and 7 (8.8%) samples were positive for EHV-1 and EHV-4, respectively, among the 79 samples collected. In addition, 5 (6.3%) samples (2 nasal swabs and 3 abortion samples) were simultaneously infected with both viruses. Interestingly, the detection rates of EHV infections during the 4-year study period were very constant (ranging from 45.1% to 46.1%). Nevertheless, the detection rates of EHV-1 and EHV-4 among aborted mares were significantly higher than their detection rates in horses affected by respiratory disease (Chi square value = 8.97 significant at *p* < 0.05). Additionally, detection rates for EHV-1 in nasal swab and abortion samples were significantly higher than those of EHV-4 (Table 3).

### 3.2. Sequencing and Phylogenetic Analysis

**Figure 1:**
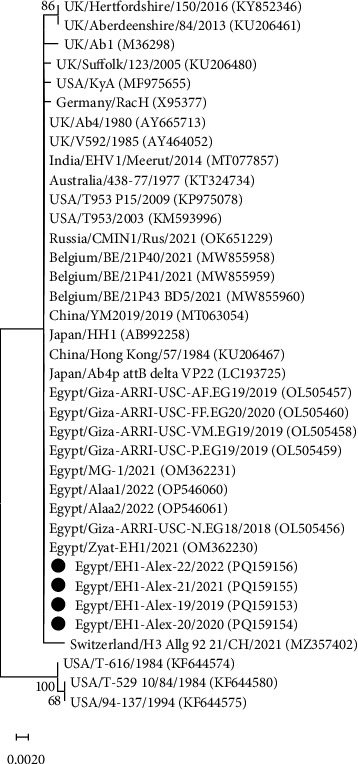
Maximum likelihood phylogenetic tree based on partial gB nucleotide sequences of EHV-1 strains. Evolutionary analysis was conducted by MEGA11 software using the Tamura–Nei model. Numbers next to branches indicate bootstrap support levels (1000 replicates). Black circles indicate the strains identified in the present study.

**Figure 2:**
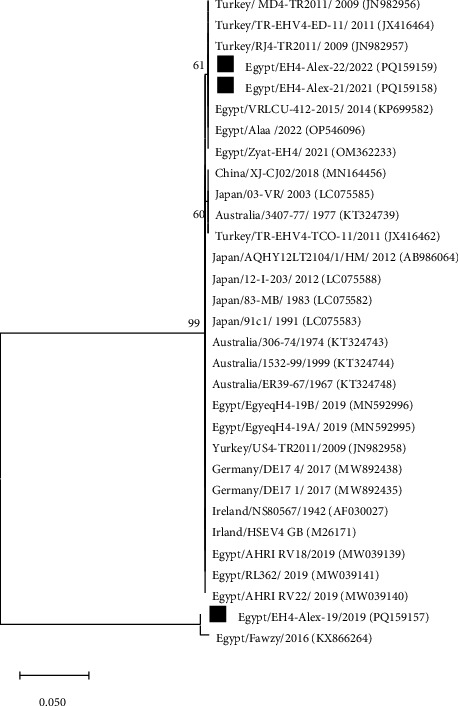
Maximum likelihood phylogenetic tree based on partial gB nucleotide sequences of EHV-4 strains. Evolutionary analysis was conducted by MEGA11 software using the Tamura–Nei model. Numbers next to branches indicate bootstrap support levels (1000 replicates). Black squares indicate the strains identified in the present study.

The gB gene was partially sequenced using PCR amplicons from four EHV-1 and three EHV-4 isolates. The resulting EHV-1 and EHV-4 gB sequences were deposited in the GenBank database with accession numbers PQ159153-PQ159156 and PQ159157-PQ159159, respectively (Table 4). Sequence alignments and phylogenetic analysis were conducted with the corresponding gB gene sequences available in the GenBank database. The EHV-1 strains (EH1-Alex-19, EH1-Alex-20, EH1-Alex-21, and EH1-Alex-22) displayed 100% nucleotide identity to each other and also to EHV-1 reference strains from Egypt and other countries, including USA, UK, Japan, India, Australia, Russia, China, and Belgium. Phylogenetic study of the nucleotide sequences identified two different clusters. The first cluster included the EHV-1 strains investigated here along with other EHV-1 reference strains obtained from horses. Meanwhile, the second cluster included EHV-1 reference strains recovered from zebra (T616), onager (T-529), and Thomson's gazelle (94–137) (Figure 1). On the other hand, the EHV-4 strains (EH4-Alex-19, EH4-Alex-21, and EH4-Alex-22) shared 76%–100% nucleotide identity and 99.3%–100% amino acid identity. Upon phylogenetic analysis of the nucleotide sequences, the EH4-Alex-19 strain was included within a distinct cluster along with the previously identified Egyptian strain Fawzy/2016 (99.3% nucleotide identity). Meanwhile, the EH4-Alex-21 and EH4-Alex-22 strains (100% nucleotide identity) were clustered with other Egyptian EHV-4 strains (99.7%–100% nucleotide identity) and EHV-4 reference strains from different countries (99.1%–100% nucleotide identity) (Figure 2). Alignment analysis of the deduced amino acid sequences of EHV-4 strains revealed that the EH4-Alex-19 and Fawzy/2016 strains possessed just one amino acid substitution (G753E) in comparison to other EHV-4 Egyptian strains.

## 4. Discussion

EHVs are ubiquitous and represent a serious threat to equine health and welfare. Despite this, little attention has been paid to determine the extent to which they have affected Egypt's equestrian industry. So yet, just a few studies have been conducted on horse populations. However, numerous infections with different EHVs, particularly EHV-1 and EHV-4, have been described in these studies [13,14,16,21]. In addition, the GenBank database still has limited sequence information about the emerging EHV-1 and EHV-4 strains in Egypt.

The present study was the first to genetically characterize the emerging EHV-1 and EHV-4 among horse populations in Alexandria province, Egypt. During the period 2019–2022, a total of 54 nasal swabs and 25 aborted fetal tissues were collected from horses displaying symptoms suggestive of EHV infection. A pan-herpesvirus-nested PCR was employed for virus detection owing to its superior sensitivity and specificity over virus isolation and serological techniques [3,9,22,23]. EHV DNA was successfully identified in 38.8% and 60% of the tested nasal swabs and abortion samples, respectively. Other studies [16,21] have also employed the same PCR assay to explore the molecular prevalence of EHVs in different geographic regions of Egypt. Similarly, these studies have revealed the presence of EHV DNA in 40.3%–54% and 50%–60% of the nasal swabs and abortion samples, respectively.

Although the proportions of EHV-positive cases were nearly similar during the 4-year study period, the detection rates of EHV-1 in nasal swabs and abortion specimens were significantly higher than those of EHV-4 (Table 3). In addition, none of the 2020 samples were positive for EHV-4. Previous studies from Egypt [14–16,21], Turkey [24], and the UK [25] have also documented that EHV-1 is more frequently detected than EHV-4. In contrast, the EHV-4 detection rates were higher than EHV-1 in other reports [13,23].

The major role of EHV-1 in inducing abortion in pregnant mares is well known worldwide [5] and in Egypt [26–28], whereas the contribution of EHV-4 remains less well documented [16,29]. Out of the 25 samples collected from abortion cases in the current study, 9 (36%) and 3 (12%) samples were positive for EHV-1 and EHV-4, respectively. Consistent with our findings, other Egyptian studies [16,21] have recorded the prevalence of EHV-1 and EHV-4 in 40%–50% and 10% of abortion samples, respectively. Another study [29], in contrast, has demonstrated an extremely high prevalence (80%) of EHV-4 in aborted fetal samples; meanwhile, EHV-1 was not detected in any sample.

Additionally, in the present study, 5 (6.3%) samples (2 nasal swabs and 3 abortion samples) were simultaneously infected with both EHV-1 and EHV-4. Concurrent infections with multiple EHVs have also been reported in other studies in Egypt [13,16,21] and worldwide [30,31]. The incidence of such infections should always be considered during surveillance studies to provide a clear picture of the epidemiological situation of EHVs in Egypt.

The gB gene plays a key role in the early events of virus–cell interactions, as well as representing a prominent target for virus-neutralizing antibodies and other immune responses [12,32]. Thus, the gB gene has been employed in most molecular genetic investigations [33]. In this study, four EHV-1 and three EHV-4 strains were characterized based on the gB gene partial sequence analysis (Table 4). Interestingly, the four EHV-1 strains (GenBank accession numbers PQ159153- PQ159156) were 100% identical to one another and also to EHV-1 reference strains reported in Egypt during the period 2018–2022, as well as in various countries over the past 5 decades (Figure 1). These findings were not surprising, considering that gB gene is the most highly conserved region in the herpesvirus genome [34].

On the other hand, the three EHV-4 strains (GenBank accession numbers PQ159157-PQ159159) shared 76%–100% and 99.3%–100% identity in their nucleotide and amino acid sequences, respectively. Also, they were grouped into 2 distinct clusters based on phylogenetic analysis of their nucleotide sequences (Figure 2). The 2019 EHV-4 strain (EH4-Alex-19) displayed a close relatedness (99.3% nucleotide identity) to the Fawzy/2016 strain, which was isolated during an abortion outbreak among Arabian mares in 2016 in Egypt [35]. Meanwhile, the 2021–2022 EHV-4 strains (EH4-Alex-21 and EH4-Alex-22) were 100% identical to each other and also to EHV-4 reference strains reported in Egypt during 2014, 2021, and 2022, as well as in Turkey during 2009 and 2011. Interestingly, the deduced amino acid sequence alignment revealed that the divergent EHV-4 strains (EH4-Alex-19 and Fawzy/2016) possessed just one amino acid substitution compared to all EHV-4 strains reported in Egypt. It has been suggested that even a single amino acid change within the highly conserved residues can significantly alter the pathogenicity of a herpesvirus. For example, the substitution of asparagine (Asn, N) for aspartic acid (Asp, D) at position 752 of the viral DNA polymerase is strongly connected to the neuropathogenic capacity of herpesviruses [36,37].

This study concludes that EHV-1 and, to a lesser extent, EHV-4 are highly prevalent in the examined horse populations in Egypt, with outbreaks of abortion and respiratory disorders which reflect substantial manifestations of these viruses. Further surveillance and genetic profiling of EHV field strains will undoubtedly provide invaluable insight into the epidemiological status of EHVs in Egypt and help to underpin a successful prevention strategy.

## Figures and Tables

**Table 1:** Consensus primers used in pan-herpesvirus-nested PCR assay.

PCR round	Primer	Sequence 5′-3′	Product size (bp)	Reference
First round	DFA-F	GAYTTYGCNAGYYTNTAYCC	215–315	[18]
	ILK-F	TCCTGGACAAGCAGCARNYSGCNMTNAA		
	KG1-R	GTCTTGCTCACCAGNTCNACNCCYTT		
Second round	TGV-F	TGTAACTCGGTGTAYGGNTTYACNGGNGT		
	IYG-R	CACAGAGTCCGTRTCNCCRTADAT		

**Table 2:** Oligonucleotide primers used in the amplification of the gB gene of EHV-1 and EHV-4.

Virus	Primer sequence	Product size (bp)	Reference
EHV-1	F: 5′-CACTTCCATGTCAACGCACT-3′	869	[14]
	R: 5′-TCGACTTTCTTCTCGGTCCA-3′		

EHV-4	F: 5′-TATTGTTTCCGCCACTCTTGACG	508	[19]
	R: 5′-GTAGAATCGGAGGGCGTGAAGC-3′		

**Table 3:** Detailed PCR results of the EHV-1 and EHV-4 detection in clinical samples.

Year	No. of samples	EHV-positive samples								
		Total positive samples (%)	Nasal swabs				Placenta and fetal lung			
			No. of positive samples	EHV-1	EHV-4	Both viruses	No. of positive samples	EHV-1	EHV-4	Both viruses
2019	24	11 (45.8%)	6/16	4	2	0	5/8	3	1	1
2020	11	5 (45.4%)	3/9	3	0	0	2/2	2	0	0
2021	13	6 (46.1%)	4/11	3	1	0	2/2	1	0	1
2022	31	14 (45.1%)	8/18	5	1	2	6/13	3	2	1
Total (%)	79 (100%)	36 (45.5%)	21/54^∗^ (38.8%)	15 (27.7%)	4 (7.4%)	2 (3.7%)	15/25^#^ (60%)	9 (36%)	3 (12%)	3 (12%)

^∗^Significant difference (chi square value = 15.077 significant at *p* < 0.01) among detection rates of EHV-1, EHV-4, and both viruses in the collected nasal swabs.

^#^Significant difference (chi square value = 10.11 significant at *p* < 0.05) among detection rates of EHV-1, EHV-4, and both viruses in the collected placenta and fetal lung samples.

**Table 4:** Details of the EHV-1 and EHV-4 strains identified in the present study.

Disease form	Sample type	Virus	Strain ID	Year of isolation	GenBank accession no
Respiratory disease	Nasal swab	EHV-1	EH1-Alex-19	2019	PQ159153
Abortion	Placenta and fetal lung	EHV-1	EH1-Alex-20	2020	PQ159154
Respiratory disease	Nasal swab	EHV-1	EH1-Alex-21	2021	PQ159155
Abortion	Placenta and fetal lung	EHV-1	EH1-Alex-22	2022	PQ159156
Abortion	Placenta and fetal lung	EHV-4	EH4-Alex-19	2019	PQ159157
Respiratory disease	Nasal swabs	EHV-4	EH4-Alex-21	2021	PQ159158
Respiratory disease	Nasal swabs	EHV-4	EH4-Alex-22	2022	PQ159159

## Data Availability

All data that support the findings of this study are present in the research article.
